# Race, Ethnicity, and Social Determinants of Health in PICU Mode of Death: Single-Center Retrospective Cohort Study

**DOI:** 10.1097/CCE.0000000000001366

**Published:** 2026-01-09

**Authors:** Amanda Alladin, Brent Pfeiffer, Paulo Nino, Sabine Mosal, Michael Nares, Monica Alba-Sandoval, Juan Pablo Solano, Barry Gelman, G. Patricia Cantwell, Asumthia Jeyapalan

**Affiliations:** 1 University of Miami Miller School of Medicine, Department of Pediatrics, Division of Pediatric Critical Care Medicine, Holtz Children’s Hospital, Miami, FL.; 2 University of Miami Institute for Bioethics and Health Policy, Miami, FL.; 3 Jackson Memorial Hospital, Holtz Children’s Hospital, Pediatric Critical Care Medicine Fellowship Program, Miami, FL.

**Keywords:** death, ethnicity, pediatric intensive care unit, racial groups, social determinants of health

## Abstract

**IMPORTANCE:**

Black and Hispanic/Latino patients are underrepresented in pediatric mode of death (MOD) studies. Although significant disparities have been reported, the associations of MOD with patient-level social determinants of health (SDOH) and the Child Opportunity Index (COI) are unknown.

**OBJECTIVES:**

To investigate associations between PICU MOD, race and ethnicity, SDOH, and COI.

**DESIGN, SETTING, AND PARTICIPANTS:**

Retrospective, single-center cohort study at a twenty-four-bed PICU within a large safety-net, public hospital, including all PICU deaths between January 2010 and December 2019.

**MAIN OUTCOMES AND MEASURES:**

We examined MOD by race and ethnicity, COI, and SDOH, including preferred language, health insurance, single-parent household status, parental occupation, and healthcare barriers abstracted from medical records. MOD was categorized as limitation of artificial life-sustaining therapies/technology (LOT) or withdrawal of artificial life-sustaining therapies/technology (WOT), failed resuscitation (FR), and death by neurologic criteria (DNC).

**RESULTS:**

Of 238 deaths, Black non-Hispanic/Latino patients comprised 42% (*n* = 100), Hispanic/Latino patients of all races 35% (*n* = 83), and White non-Hispanic/Latino patients 19% (*n* = 46). LOT/WOT was the predominant MOD (75%, *n* = 174; LOT = 109, WOT = 65). Racial and ethnic groups showed significant differences in COI, SDOH, and healthcare barriers. Despite this, there were no significant differences in MOD by Race and Ethnicity, SDOH, or healthcare barriers. Median COI was lower for DNC compared to LOT and WOT, and for FR compared with WOT. However, when examined within individual racial and ethnic groups, there was no difference in median COI between FR, LOT, and WOT.

**CONCLUSION AND RELEVANCE:**

We found no differences in MOD by Race and Ethnicity, SDOH, or barriers. Median COI was lower for FR compared with WOT. This suggests that COI, as opposed to race and ethnicity, may play a role in pursuing or forgoing resuscitation at end-of-life. This study adds to the examination of pediatric healthcare disparities at end-of-life by including SDOH and COI data in MOD analysis.

**KEY POINTS**

**Question:** Are there associations between race and ethnicity, social determinants of health (SDOH), the Child Opportunity Index (COI), and PICU mode of death (MOD)?

**Findings:** In a cohort of predominantly Black and Hispanic/ Latino patients, PICU MOD did not vary by race and ethnicity, patient-level SDOH, or healthcare barriers. Median COI was lower in the death by neurologic criteria group compared with the withdrawal and limitation of technology/therapy groups, and lower for the failed resuscitation group compared with the withdrawal of technology/therapy group.

**Meaning:** Disparities in PICU mode of death may be associated with COI, as opposed to race and ethnicity.

Modes of death (MOD) in the PICU have been studied in the United States and in other developed and developing countries (1–4). Although it seems established that the most frequent modes of pediatric death in the United States are related to limitation or withdrawal of artificial life-sustaining therapies/technology, the impact of race, ethnicity, and social determinants of health (SDOH) on pediatric MOD has not been fully elucidated.

Robust adult studies describe concerning racial, ethnic, and socioeconomic disparities in the ICU, such as a lower likelihood of Black and Hispanic patients choosing withdrawal or limitation of technology, and higher treatment intensity at end-of-life for patients with lower education and income levels (5–7). Pediatric MOD studies examining race and ethnicity are limited with older studies reporting the possibility of Black patients being less likely to have limitation of technological support or more likely to experience cardiopulmonary resuscitation before death (8, 9). Other related pediatric studies report a higher intensity of care for Black and Hispanic children at end-of-life in specific clinical conditions (10, 11). These healthcare disparities are a critical area of concern in pediatric and adult medicine. Addressing this requires inclusion of historically underserved racial and ethnic groups in clinical research (12, 13).

Our study examines MOD over a 10-year period at a quaternary-level PICU serving a high proportion of patients who identify as historically minoritized racial and ethnic groups. These groups have been underrepresented in most pediatric modes of death studies in the United States. In addition, previous studies have not included detailed, individualized data on patient-level SDOH, limiting meaningful exploration of healthcare inequities (14). In our study, we sought to analyze correlations between race and ethnicity, and patient-level SDOH with MOD. Markers of established SDOH, Child Opportunity Index (COI), healthcare barriers as well as other related variables potentially impacting end-of-life were recorded, and associations examined (15, 16).

## METHODS

This study was approved by the University of Miami Institutional Review Board (IRB Number 20201301, 11/2020). Study procedures were conducted in accordance with institutional and federal ethical standards and the Helsinki Declaration of 1975. This is a single-center observational cohort study from January 2010 to December 2019, at a quaternary-level PICU. Data were collected via retrospective chart review for all patients who died in the PICU over the study period. No deaths were excluded. Each patient had MOD classified based on review of documentation and orders before death. Four modes were identified: failed resuscitation (FR), limitation of artificial life-sustaining therapies/technology (LOT), withdrawal of artificial life-sustaining therapies/technology (WOT), or death by neurologic criteria (DNC). FR was defined as initiation of cardiopulmonary resuscitation without return of spontaneous circulation. LOT was defined as placement of a “do not resuscitate” or “do not intubate” order or a documented decision not to escalate therapies despite clinical deterioration. WOT was defined as active de-escalation of technology/therapies, such as discontinuation of mechanical ventilation or vasoactive medication, and/or extubation with the expectation of death. MOD was determined by the decision at the time of expiration. Given that both LOT and WOT are considered a form of “forgoing” and are morally and ethically equivalent, they were analyzed together as well as individually where possible (17, 18).

Patient’s self-reported race and ethnicity were recorded from the demographics section of the electronic medical record, as well as Social Worker interviews, and documentation. Race and ethnicity are typically documented at registration using the U.S. Census Bureau and the Agency for Healthcare Research and Quality classifications (19, 20). Race was categorized as American Indian or Alaska Native, Asian, Black or African American, White, Native Hawaiian or Pacific Islander, or Other. Ethnicity was categorized as Hispanic or Latino or Not Hispanic or Latino. Groups were analyzed using a combination of racial and ethnic identity: Black Non-Hispanic or Latino, Hispanic or Latino of any race, and White Non-Hispanic or Latino.

SDOH and potential healthcare barriers were identified using Healthy People 2030 Social Determinants of Health Categories (**SDC Table 1**, https://links.lww.com/CCX/B594) (16). Health insurance information was grouped into government, private, self-pay, and international payor types. Preferred language, single-parent or caregiver household status, and parental occupation were also collected. Potential barriers were derived from Social Worker/Case Manager narratives, including current and prior admissions. These included financial, housing, transportation, legal/immigration, health literacy, mental health, and parental/guardian physical health concerns. Number of barriers was categorized into 0, 1, or greater than or equal to 2 according to the 50th percentile of the dataset. Additional demographic and clinical data collected included age at death, sex assigned at birth, PICU length of stay, presence of a palliative care consult, and etiology of primary illness. Primary illness was classified as acute or complex chronic per the system pioneered by Feudtner et al (21). A complex chronic condition is defined as one “expected to last at least 12 months … involve several different organ systems or 1 organ system severely enough to require specialty pediatric care/hospitalization in a tertiary care center” (21). Acute presentations of expected complex chronic conditions were categorized as chronic.

The COI is a composite index of children’s neighborhood opportunity based upon U.S. zip code data (22). The COI 3.0 is an amalgam of Education, Health and Environment, and Social and Economic data measuring 44 different neighborhood conditions, resulting in a single metric that can be compared. Published literature, including COI measurements, has been instrumental in demonstrating structural racism and inequity by highlighting how different neighborhoods support child health and development (23). We obtained the COI 3.0 weighted for zip codes, and each patient’s zip code was matched to a corresponding COI 3.0 measurement (**SDC Data** & **Software**, https://links.lww.com/CCX/B594). Our PICU is located within a very low COI zip code, in geographic proximity to multiple low-income populations, medically underserved, and health professional shortage areas (22). Parental occupation was classified using COI definitions for “high skill occupations” (SDC Data & Software https://links.lww.com/CCX/B594).

We used R and RStudio with the following packages (tidyverse, gt, gtsummary, and ggsignif) to clean, combine, explore, visualize, and summarize our datasets. (SDC Data & Software, https://links.lww.com/CCX/B594) We filtered the COI dataset for the year 2020, following COI use guidelines, joining COI observations with our dataset based on matching zip codes. International patients did not have a COI. Most data presented is descriptive except for assessing the modes of death by race and COI numbers. A Fisher exact test or a Kruskal-Wallis rank sum test was applied, respectively, to determine the mode of death differences between race and ethnicity and COI. For the investigation of the COI by race and ethnicity, we applied an analysis of variance and Tukey Honestly Significant Difference (HSD) to determine significant differences between multiple groups.

## RESULTS

### General Characteristics

We recorded 238 deaths over the 10-year study period. Race and ethnicity data are summarized in **Table 1**, with demographic and clinical variables by MOD. Patients identifying as Black Non-Hispanic or Latino (hereafter referred to as Black) were the largest racial and ethnic group represented (42%, *n* = 100). Patients identifying as White Hispanic or Latino, and Black Hispanic or Latino were the second largest group (35%, *n* = 83) (hereafter referred to as Hispanic/Latino). Only three patients identified as Black Hispanic/Latino, limiting in-depth analysis for this subgroup. Patients identifying as White Non-Hispanic or Latino (hereafter referred to as White) were the third largest racial and ethnic group (19%, *n* = 46). Most patients had complex chronic conditions (78%, *n* = 186), and proportions of acute vs. chronic conditions were similar across the three largest racial and ethnic groups (**SDC Fig. 1**, https://links.lww.com/CCX/B594). Acute presentations were more prevalent in DNC vs. LOT, WOT, and FR (Table 1; and **SDC Table 2**, https://links.lww.com/CCX/B594). Most patients came from dual-parent/caregiver households (68%), and the majority reported English as their preferred language (72%), whereas 21% reported Spanish as their preferred language. The most common categories of primary illness were oncologic, gastrointestinal, cardiac, trauma, and genetic, constituting 77% of the cohort (**SDC Fig. 2**, https://links.lww.com/CCX/B594). The overall mortality rate for the study period was 3.9%. Proportions of PICU deaths and PICU admissions by race and ethnicity were similar over the study period. These proportions approached the 2020 U.S. Census data for our dataset’s 135 unique zip codes (**Table 2**). For the entire cohort, median PICU length of stay was 10.5 days (interquartile range [IQR] 24 d), and 37% (87/238) were referred to palliative care.

**TABLE 1.** General Characteristics by Mode of Death

Variable	*n*	Limitation or Withdrawal of Artificial Life-Sustaining Therapies, *n* = 174^a^	Failed Resuscitation, *n* = 33^a^	Death by Neurologic Criteria, *n* = 31^a^	*p* ^b^
Race and ethnicity	238				0.3
Black		68 (39%)	14 (42%)	18 (58%)	
Hispanic or Latino		59 (34%)	16 (48%)	8 (26%)	
White		39 (22%)	3 (9.1%)	4 (13%)	
Asian		4 (2.3%)	0 (0%)	0 (0%)	
Unknown		4 (2.3%)	0 (0%)	1 (3.2%)	
Age (yr)	238	7.0 (1.0, 15.0)	10.0 (1.0, 16.0)	6.0 (1.0, 13.0)	0.5
Sex assigned at birth	238				0.8
Female		74 (43%)	14 (42%)	11 (35%)	
Male		100 (57%)	19 (58%)	20 (65%)	
Acute vs. chronic illness	238				< 0.001
Acute		24 (14%)	7 (21%)	21 (68%)	
Chronic		150 (86%)	26 (79%)	10 (32%)	
Payor	238				0.070
Government		125 (72%)	21 (64%)	23 (74%)	
Private		31 (18%)	6 (18%)	3 (9.7%)	
International		15 (8.6%)	4 (12%)	1 (3.2%)	
Self pay		3 (1.7%)	2 (6.1%)	4 (13%)	
Preferred language	238				0.7
English		125 (72%)	21 (64%)	26 (84%)	
Spanish		38 (22%)	9 (27%)	4 (13%)	
Haitian Creole		7 (4.0%)	2 (6.1%)	1 (3.2%)	
Other		4 (2.3%)	1 (3.0%)	0 (0%)	
Parents/caregiver in household^c^	235				0.7
Dual		121 (70%)	23 (74%)	19 (63%)	
Single		53 (30%)	8 (26%)	11 (37%)	

a *n* (%); median (Q1, Q3).

b Fisher exact test; Kruskal-Wallis rank sum test; Pearson *χ*^2^.

c Patients excluded due to wards of the state or not living with parents.

**TABLE 2.** PICU Deaths, Admissions, and 2020 U.S. Census Data by Race and Ethnicity (January 2010 to December 2019)

Race and Ethnicity	Death (%)	Admit (%)	Census (%)
Black	42	44	34
Hispanic/Latino	35	35	41
White	19	18	23
Other	5	3	NA
Asian	2	1	2

Census (%) represents mean proportions of race and ethnicity from 135 unique zip codes in our dataset.

### Modes of Death by Race and Ethnicity

MOD was not statistically different when compared among the racial and ethnic groups within our cohort (Table 1; and **SDC Table 2**, https://links.lww.com/CCX/B594). LOT was the most prevalent MOD among the three largest racial and ethnic groups, followed by WOT (SDC Table 2, https://links.lww.com/CCX/B594). There was no difference in MOD for the three largest racial and ethnic groups when LOT and WOT were separated (SDC Table 2, https://links.lww.com/CCX/B594). Due to the small number of patients in the Asian and Unknown groups, we were unable to analyze differences in MOD with LOT and WOT separated.

### Modes of Death by COI and Race and Ethnicity

**Figure 1.**
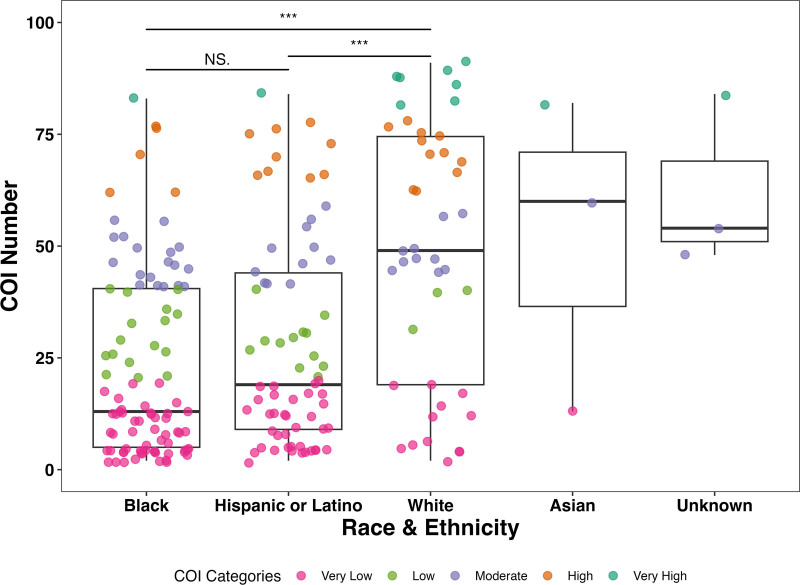
Distribution of the nationally normalized Child Opportunity Index (COI) numbers associated with the patient’s zip code separated by race and ethnicity. Black and Hispanic or Latino patients came from zip codes with significantly lower COI numbers than White patients. An all pairwise analysis by analysis of variance and Tukey Honestly Significant Differene (HSD); *** is a *p* value < 0.001. A majority of Black and Hispanic or Latino patients came from zip codes falling within Low (*green*) or Very Low (*pink*) COI Categories. Most White patients fall within Moderate (*purple*), High (*orange*), or Very High (*blue*) COI categories. NS = non-significant.

Analysis of COI data showed that 71% (*n* = 71) of Black patients and 58% (*n* = 48) of Hispanic/Latino patients lived in zip codes categorized as Very Low or Low COIs (**Fig. 1**). In comparison, only 32% (*n* = 28) of White patients lived in Low and Very Low COI zip codes. When assessing COI scores among the three largest racial and ethnic groups, median (IQR) COI scores were significantly greater for White individuals (49, IQR 55.5) than Hispanic/Latino (19, IQR 35) or Black (13, IQR 35.5) groups (Fig. 1).

**Figure 2.**
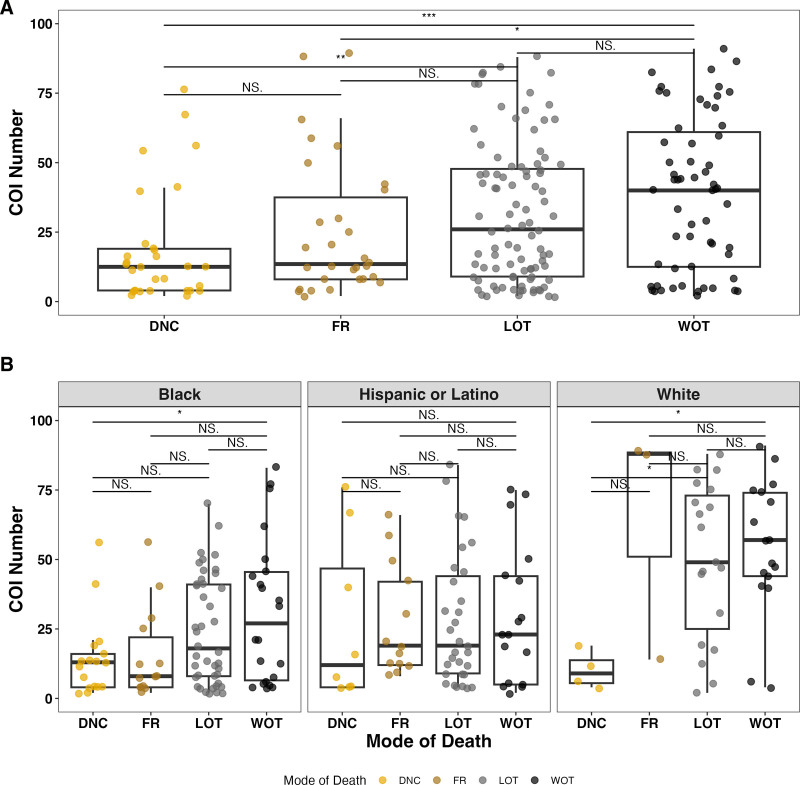
Mode of death by Child Opportunity Index (COI). **A**, Distribution of the nationally normalized COI numbers associated with the patient’s zip code separated by mode of death. The median COI is lower in death by neurologic criteria (DNC) compared with limitation of artificial life-sustaining therapies/technology (LOT) and withdrawal of artificial life-sustaining therapies/technologies (WOT), as well as failed resuscitation (FR) compared with WOT. An all pairwise analysis by analysis of variance (ANOVA) and Tukey Honestly Signficant Difference (HSD); *, **, and *** are *p* < 0.05, *p* < 0.01, and *p* < 0.001, respectively. **B**, Distribution of the nationally normalized COI numbers and mode of death disaggregated by race and ethnicity. For Black patients, median COI is lower for DNC compared with WOT. There is no difference in COI between LOT, WOT, FR, and DNC for Hispanic/Latino patients. For White patients, median COI is lower for DNC compared with LOT and WOT. An all pairwise analysis within race and ethnicity by ANOVA and Tukey Honestly Significant Difference (HSD); * is *p* < 0.05. NS = non-significant.

For the overall cohort, median COI scores were lower for DNC (12.5, IQR 15) compared with LOT (26, IQR 38.7) and WOT (40, IQR 48.5), as well as FR (13.5, IQR 29.5) compared with WOT (**Fig. 2*A***). Median COI scores by MOD were also examined individually for each racial and ethnic group. We found no significant differences in median COI among LOT, WOT, FR, and DNC for Hispanic/Latino patients (**Fig. 2*B***). For Black patients, the median COI was lower in DNC (13, IQR 12) vs. WOT (27, IQR 39). For White patients, median COI was lower for DNC (9, IQR 8.25) compared with LOT (49, IQR 48) and WOT (57, IQR 30). There was no significant difference in median COI within any racial and ethnic group between LOT, WOT, and FR.

### Modes of Death by SDOH and Documented Healthcare Barriers

We found no statistical difference in MOD by health insurance payor, preferred language, or single-parent/caregiver household status. (Table 1; and SDC Table 2, https://links.lww.com/CCX/B594). There was no difference in LOT, WOT, and FR by the number of healthcare barriers documented (**Table 3**). DNC was excluded from analysis by the number of barriers, as 68% of this group had an acute presentation with a limited social worker narrative and insufficient documentation of healthcare barriers. Patients with complex chronic illnesses generally had more documentation from which to abstract barriers. Black and Hispanic/Latino patients were more likely to have greater than 1 barrier compared with White patients (Table 3). Black and Hispanic/Latino patients were more likely to be insured through the government compared with White patients (**Table 4**). There was significant missing data for parental occupation, limiting analysis by MOD. Of the data available, Black and Hispanic/Latino patients had a smaller percentage of parents in a high-skilled occupation compared with White patients. A higher percentage of Black and Hispanic/Latino patients experienced financial and transportation barriers compared with White patients, whereas Hispanic/Latino patients more commonly faced legal/immigration barriers compared with other groups (Table 4).

**TABLE 3.** Number of Barriers by Mode of Death (Excluding Death by Neurologic Criteria) and Race and Ethnicity

Characteristic	Mode of Death				Race and Ethnicity			
	Failed Resuscitation, *n* = 33^a^	Limitation of Artificial Life-Sustaining Therapies, *n* = 109^a^	Withdrawal of Artificial Life-Sustaining Therapies, *n* = 65^a^	*p* ^b^	Black, *n* = 100^a^	Hispanic or Latino, *n* = 83^a^	White, *n* = 46^a^	*p* ^b^
Barrier				0.4				0.025
0	10 (30%)	31 (28%)	24 (37%)		39 (39%)	18 (22%)	22 (48%)	
1	11 (33%)	24 (22%)	16 (25%)		22 (22%)	24 (29%)	11 (24%)	
2 or greater	12 (36%)	54 (50%)	25 (38%)		39 (39%)	41 (49%)	13 (28%)	

a *n* (%).

b Pearson *χ*^2^.

Barriers included housing, financial, transport, literacy, legal/immigration, mental health, and parental health.

**TABLE 4.** Payor, Parental Occupation Classification and Individual Barriers by Race and Ethnicity

Characteristic	Black, *n* = 100^a^	Hispanic or Latino, *n* = 83^a^	White, *n* = 46^a^	*p* ^b^
Variables				
Payor				< 0.001
Government	86 (86%)	58 (70%)	22 (48%)	
Private	9 (9.0%)	10 (12%)	19 (41%)	
International	3 (3.0%)	11 (13%)	3 (6.5%)	
Self pay	2 (2.0%)	4 (4.8%)	2 (4.3%)	
Parental occupation classification				0.011
High skill	14 (14%)	15 (18%)	18 (39%)	
Non-high skill	27 (27%)	29 (35%)	8 (17%)	
Unemployed	13 (13%)	11 (13%)	3 (6.5%)	
Unknown	46 (46%)	28 (34%)	17 (37%)	
Individual barriers^c^				
Housing	30 (30%)	39 (47%)	15 (33%)	
Financial	43 (43%)	44 (53%)	12 (26%)	
Transport	28 (28%)	26 (31%)	6 (13%)	
Literacy	5 (5.0%)	5 (6.0%)	0 (0%)	
Legal/immigration	15 (15%)	23 (28%)	4 (8.7%)	
Mental health	7 (7.0%)	6 (7.2%)	1 (2.2%)	
Parental health	9 (9.0%)	1 (1.2%)	3 (6.5%)	

a *n* (%).

b Fisher exact test; Pearson *χ*^2^.

c Individual Barriers are not mutually exclusive for each patient.

## DISCUSSION

In our single-center study of PICU deaths over a ten-year period, we found no significant difference in pediatric MOD among Black Non-Hispanic, White Non-Hispanic, and Hispanic/Latino patients of any race, the three largest groups in our cohort. Black Non-Hispanic and Hispanic/Latino patients have been underrepresented in previous pediatric mode of death studies but comprised the majority of our study population. We also found no significant difference in MOD by patient-level SDOH and potential healthcare barriers abstracted from individual charts. Black and Hispanic/Latino patients in our cohort came from lower COI areas compared with White patients. Although there were no differences detected in MOD by race and ethnicity, important differences in MOD were assessed by COI. A lower median COI was associated with DNC compared with LOT and WOT, as well as FR compared with WOT.

Our results seemingly contrast with racial and ethnic disparities described in older pediatric and adult literature—that Black and Hispanic/Latino patients were less likely to choose withholding and withdrawing of technology, or more likely to undergo resuscitation (6–9, 24). Indeed, a more recent study by White-Makinde et al (25) found no differences in MOD between Black and White children. At a glance, the lack of difference in MOD between racial and ethnic groups could be interpreted as potential delivery of equitable end-of-life care at our institution. Our patient population predominantly consists of Black and Hispanic/Latino patients who are insured through Medicaid. We are fortunate to have both physicians and nursing staff who reflect the diversity of the population we serve. Over the study period, our PICU physicians were 42% Hispanic/Latino and 9% Black Non-Hispanic. Aggregate PICU nursing data for the study period were unavailable but currently, there are 43% Hispanic/Latino nurses and 36% Black Non-Hispanic nurses, many of whom were employed during the study period. All our Hispanic/Latino physicians and nurses are fluent in Spanish, as are some of our non-Hispanic/Latino physicians and nurses. These unique institutional characteristics may impact the quality of end-of-life care delivered, especially for patients and families identifying as Black Non-Hispanic or Hispanic/Latino (26, 27). The racial and ethnic proportions of admissions to our PICU, as well as mean census data, are also congruent with the racial and ethnic proportions of deaths, supporting the possibility of equitable care.

Moving past this positive but superficial analysis requires consideration of factors not captured in this study. Important pediatric studies have documented significant variability in the content and conduct of end-of-life conversations, which may impact mode of death (28, 29). In 2016, Keele et al (28) documented from the large Collaborative Pediatric Critical Care Research Network, a lower likelihood of discussion of limitation or withdrawal of life-sustaining therapies for Black families. Gupta et al (29) also found that in a high mortality cohort, there was inconsistent prognostic information given to parents despite being asked to make a critical decision. We cannot definitively state that there was equity in end-of-life care as the quality, frequency, and choice architecture of discussions were not studied for our cohort. Specifically, the use of valid shared decision-making at end-of-life vs a paternalistic or nudging approach was not assessed, which could have contributed to the homogeneity of our findings. As defined by the U.S. Census Bureau, a significant proportion of our patients and families would be classified as first or second-generation foreign-born. This may have impacted the acceptance of medical recommendations and exertion of parental preferences, especially if coming from cultures where medical paternalism is more widely accepted (30). Most of our cohort resided in a low or very low COI area, which suggests lower “social capital” and possibly decreased likelihood to object to a medically recommended plan of care (31, 32).

There were differences detected in MOD by median COI. Median COI was lower in FR compared with WOT for the overall cohort. This may be aligned with contemporary adult and pediatric studies reporting associations of higher intensity of end-of-life care with lower income and education levels (11, 33, 34). However, within individual racial and ethnic groups, median COI was not statistically different between FR and LOT or WOT. This suggests that race and ethnicity were not associated with the decision to pursue or forgo resuscitation at end-of-life. For the overall cohort, patients in the DNC category had a lower median COI compared with LOT and WOT. The association of lower median COI with DNC may reflect a higher risk of trauma, suicide, and unintentional injuries in children with lower socioeconomic status or from lower COI areas (35–40). Indeed, 18 patients (58%) in the DNC group had a primary diagnosis of trauma, sudden unexplained infant death, drowning, and suicide. For both Black and White patients, the median COI was lower for DNC compared with WOT. This possibly contrasts with adult literature reporting that Black and Hispanic patients may be less likely to opt for withdrawal after a trauma diagnosis compared with White patients (24). Race and ethnicity may have inadvertently reflected deeper disparities in SDOH or health literacy. Additionally, some patients with suspected DNC and higher median COI may have undergone WOT or LOT before confirmation of DNC. The possible relationship between a higher COI and increased likelihood of WOT vs FR in pediatrics requires further exploration. Current adult literature is mixed, showing associations between withdrawal and higher socioeconomic status, but also associations between early withdrawal and uninsured status (33, 34, 41, 42).

As a quaternary referral center associated with a high-volume transplant center, our cohort predominantly consisted of patients with complex chronic conditions and included a large proportion of patients with primary oncologic disease. This may have supported LOT and WOT decisions as there may have been more time and prognostic information to facilitate goals-of-care discussions. Palliative care team involvement may impact MOD. Our palliative care team consists of a nurse coordinator, interdisciplinary teams such as child life and spiritual care, with inconsistent, voluntary physician coverage for challenging cases. As such, although 37% (87/238) of our cohort was referred to palliative care, the effect of referral could not be fully assessed due to potential variability of palliative care team member involvement. Of note, trends in the predominant MOD did not vary greatly over the study period (**SDC Fig. 3**, https://links.lww.com/CCX/B594).

This study has limitations that must be acknowledged. Institutional efforts to standardize and improve SDOH screening and documentation of patients’ self-reported race and ethnicity were conducted after our study period. Thus, some patients may have experienced misclassification of race, ethnicity, and healthcare barriers. We had few patients identifying as races and ethnicities outside of our three largest groups, limiting analysis for these patients. End-of-life care for Black Hispanic/Latino patients is an understudied area. Analysis of this subgroup was not feasible and although not ideal, these three patients were included in the Hispanic/Latino group. Our PICU has many fluent Spanish speakers readily available on staff, which may not reflect the in-person language capabilities of other centers. The COI 3.0 data represent zip code classification as of 2020; therefore, changes in COI by zip code were not assessed over time. Missing data for clinical confounders, parental occupation, and potential barriers precluded important analyses. Finally, our electronic medical record does not capture family meetings or goals-of-care conversations separately. This coupled with highly variable provider documentation, made it challenging to include end-of-life meetings and decision-making in this study. Changes in code status and goals during hospitalization were therefore unavailable for analysis. Our single-center practice and small sample size are also not adequately powered to detect differences that may be more evident in a larger data set, such as the Virtual PICU Network.

Prospective research is warranted to consider how SDOH impacts decision-making at pediatric end-of-life. Do health insurance, financial, transportation, or health literacy barriers contribute to decisions regarding forgoing resuscitation? (41) The association of lower COI with DNC suggests that this is an at-risk group requiring preventative interventions and support. Qualitative, multicenter studies are also needed to elicit perspectives of historically minoritized groups and shed light on their perceptions of the quality of supported decision-making at end-of-life (43).

## CONCLUSION

In this single-center study spanning a decade of care, we found no difference in PICU MOD when analyzed by race and ethnicity and other SDOH, although many of our Black and Hispanic/Latino patients came from neighborhoods with low or very low COI and faced more healthcare barriers compared with White patients. For the entire cohort, patients with DNC had a lower median COI compared with LOT and WOT. Median COI was also lower in FR compared with WOT for the entire cohort but when disaggregated by race, this difference was no longer observed. Thus, despite the significant differences in COI among racial and ethnic groups, race and ethnicity were not associated with forgoing or pursuing resuscitation at end-of-life. Structural and institutional racism have contributed to inequitable medical care of pediatric patients and it is incumbent upon institutions and individuals to confront this (35, 44, 45). Race and ethnicity are social constructs, yet examining these factors is crucial to assessing inequities in end-of-life care and MOD (46). Further study of end-of-life communication and care in our PICU needs to be undertaken to assess other equally important markers of equity.

## ACKNOWLEDGMENTS

We thank the nurses, respiratory therapists, and interdisciplinary staff of the Holtz Children’s Hospital PICU for providing excellent care to all our patients and their families, and Dr. Cantwell for her leadership of our team.

## Supplementary Material


