# Supplementary material for: Race, Ethnicity, and Social Determinants of Health in PICU Mode of Death: Single-Center Retrospective Cohort Study
Source: Crit Care Explor. 2026 Jan 9;8(1):e1366. doi: 10.1097/CCE.0000000000001366 (PMC12795013; doi:10.1097/CCE.0000000000001366)
Supplement: Supplementary file 1 [file cc9-8-e1366-s001.pdf]

Supplemental Digital Content (SDC):

Title:

Race, Ethnicity and Social Determinants of Health in PICU Mode of Death - Single Center Retrospective Cohort Study

Corresponding Author:

Amanda Alladin, 1611 NW 12th Avenue, Holtz Children's Hospital, ET 6006, Miami, Florida 33136. email: aalladin@med.miami.edu

Funding:

The Authors declare that there are no conflicts of interest. In addition, the Authors received no financial support for the research, authorship and/or publication of this article

Keywords:

Pediatric Intensive Care Unit; Death; Racial Groups; Ethnicity; Social Determinants of Health

Table of contents

Supplemental Digital Content (SDC) 2
SDC Tables & Figures 2
SDC - Table 1 2
SDC - Figure 1 3
SDC - Table 2 4
SDC - Figure 2 5
SDC - Figure 3 6
SDC Data & Software 7
References 8

Supplemental Digital Content (SDC)

SDC Tables & Figures

SDC - Table 1

Table 1: SDOH Definitions Table

Social Determinants of Health Categories<br>Social Determinants of Health Literature Summaries - Healthy People 2030	
	Expanded description of Barriers & Variables
Economic Stability:	
Parental Occupation	As per COI 3.0 definitions for high skilled occupations
Financial Barrier	Documentation of fundraising, Financial assistance from family members, Unemployment, Need for food stamps, Inability to cover costs associated with care plans
Transportation Barrier	Documented as lacking transportation in notes Family does not own a vehicle or no reliable access to transport, No access to carseats, Parents do not have their driver's license
Health Care Access and Quality:	
Health Literacy Barrier	Documentation of limited literacy or illiteracy in Social Worker notes, Severe difficulty understanding plans of care
Health Insurance Type	Government, Private, International, and Self Pay
Neighborhood and Built Environment:	
Housing Barrier	Not living in own home, Reliance on friends or family, Staying at charity provided housing because nowhere else to go, Out of state or city and cannot afford housing locally
Social and Community Context:	
Preferred Language	Preferred language of parents or legal decision maker if patient was < 18yrs, Preferred language of patient used if they were > 18yrs of age
Single parent or caregiver household status	Living with only one parent/guardian, Second parent not significantly involved, Including if one parent not residing in the same country as the patient
Legal Barrier	DCF involvement, Incarceration in one or both parents, Legal case pending, Unlawful immigration status, Reported domestic violence between parents or in the household
Mental Health Barrier	Parent or child dealing with psychiatric diagnosis
Parental Health Barrier	Physical health condition of parent

*Notes did not specifically assess for food insecurity during the study period. Documentation of use of food stamps was categorized as a financia barrier

Source - https://health.gov/healthypeople/priority-areas/social-determinants-health/literature-summaries

SDC - Figure 1

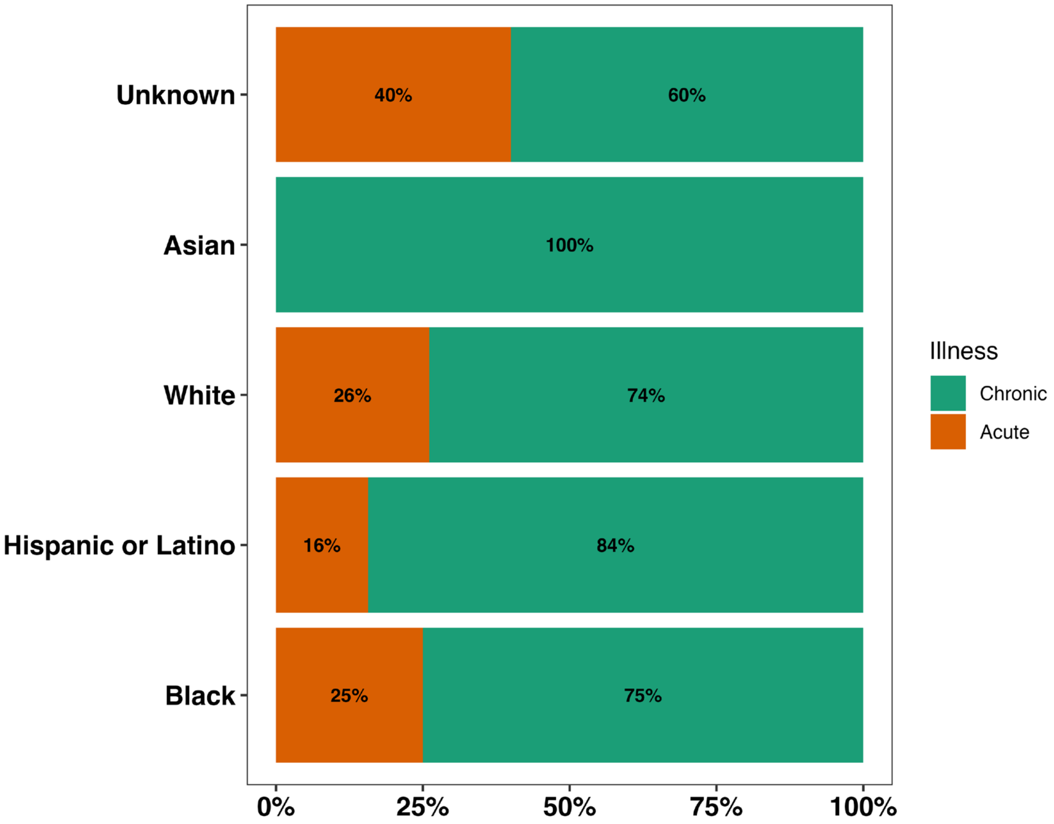

Figure 1: Proportions of acute versus chronic complex conditions by Race & Ethnicity.

SDC - Table 2

Table 2: General Characteristics by Mode of Death: LOT/WOT Separated

Variable	N	DNC N = 29[1]	FR N = 30[1]	LOT N = 103[1]	WOT N = 59[1]	p-value[2]
**Race & Ethnicity**	221					0.2
Black		17 (59%)	13 (43%)	46 (45%)	21 (36%)	
Hispanic or Latino		8 (28%)	14 (47%)	36 (35%)	21 (36%)	
White		4 (14%)	3 (10%)	21 (20%)	17 (29%)	
**Age (years)**	221	6.0 (1.0, 12.0)	5.5 (1.0, 16.0)	7.0 (1.0, 16.0)	5.0 (1.0, 15.0)	0.2
**Sex Assigned At Birth**	221					0.2
F		9 (31%)	12 (40%)	37 (36%)	30 (51%)	
M		20 (69%)	18 (60%)	66 (64%)	29 (49%)	
**Acute vs Chronic illness**	221					<0.001
Acute		19 (66%)	7 (23%)	10 (9.7%)	13 (22%)	
Chronic		10 (34%)	23 (77%)	93 (90%)	46 (78%)	
**Payor**	221					
Government		22 (76%)	19 (63%)	74 (72%)	46 (78%)	
International		1 (3.4%)	4 (13%)	8 (7.8%)	3 (5.1%)	
Private		3 (10%)	5 (17%)	20 (19%)	8 (14%)	
Self Pay		3 (10%)	2 (6.7%)	1 (1.0%)	2 (3.4%)	
**Preferred Language**	221					0.8
English		24 (83%)	19 (63%)	75 (73%)	42 (71%)	
Hatian Creole		1 (3.4%)	2 (6.7%)	5 (4.9%)	2 (3.4%)	
Spanish		4 (14%)	9 (30%)	23 (22%)	15 (25%)	
**Parents/Caregiver in Household**	221					0.5
Single		11 (38%)	8 (27%)	36 (35%)	15 (25%)	
Dual		18 (62%)	22 (73%)	67 (65%)	44 (75%)	

[1] n (%); Median (Q1, Q3)

[2] Pearson's Chi-squared test; Kruskal-Wallis rank sum test; NA; Fisher's exact test

*Due to small numbers in these categories, "Asian" and "Unknown" patients were excluded, in order to conduct statistical analysis. This reduced number of observations from 238 to 221. Three patients are also excluded from the "Parent/Caregiver in Household" category as they were wards of the state or not living with parents.

SDC - Figure 2

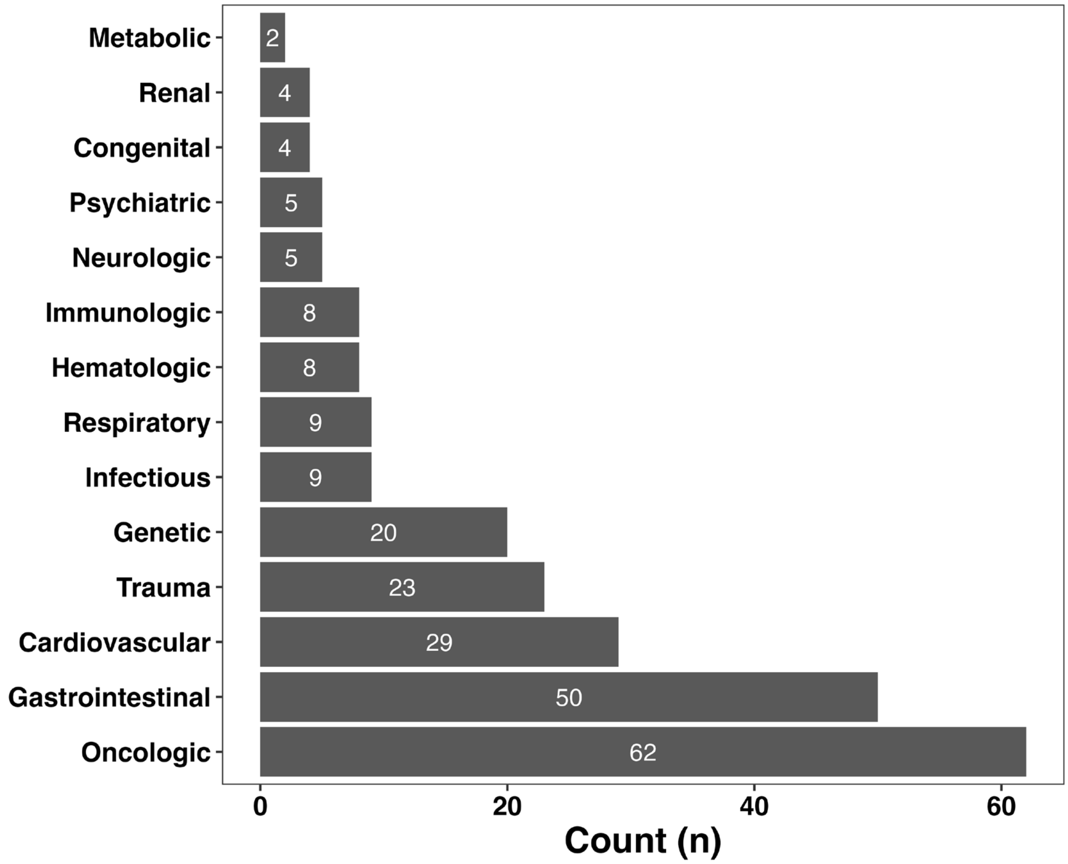

Figure 2: Deaths categorized by etiology of primary illness.

SDC - Figure 3

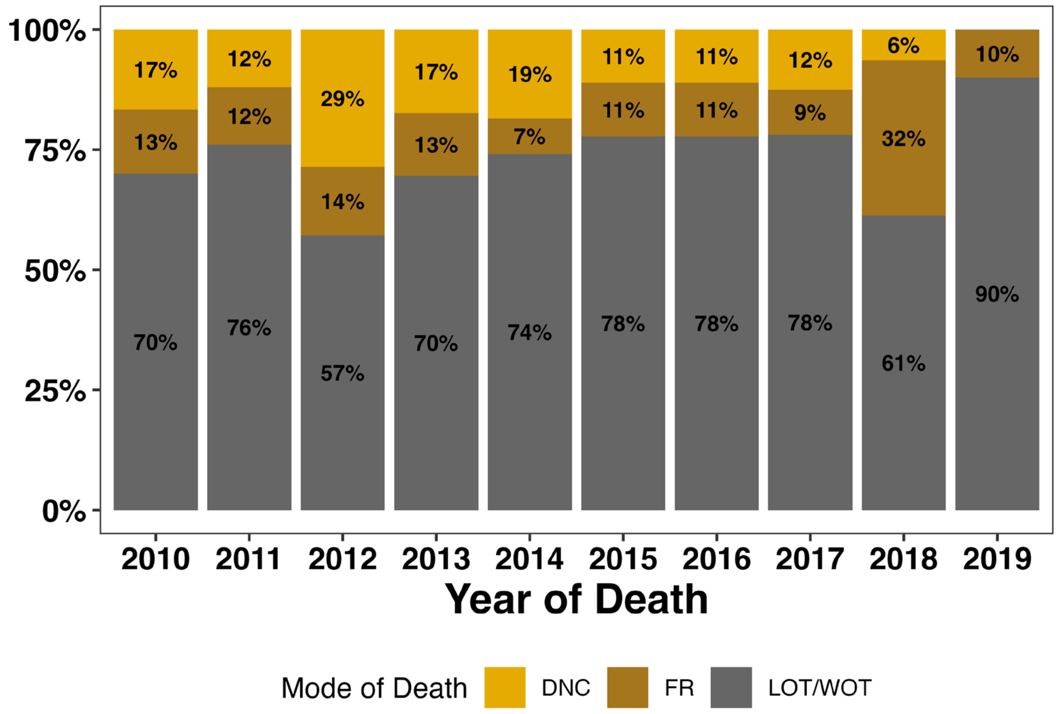

Figure 3: Mode of death proportions by year between Jan 2010 to Dec 2019. The predominant mode of death each year was LOT/WOT.

SDC Data & Software

The COI is a composite index of children's neighborhood opportunity based upon the US government census tracts data.(1) The COI is an amalgam of Education, Health & Environment, and Social & Economic data measuring 44 different neighborhood conditions, resulting in a single metric that can be compared. Published literature including COI measurements have been instrumental in demonstrating structural racism and inequity, by highlighting how different neighborhoods support child health and development.(2) We obtained the COI 3.0 weighted for zip codes, according to US census tracts. Each patient's zip code was matched to a corresponding COI 3.0 measurement, see supplemental section for software and data references.(3) Our PICU is located within a very low COI zip code, in geographic proximity to multiple low-income populations, medically underserved and health professional shortage areas.(1,4–6) Parental occupation was classified using COI definitions for "high skill occupations".(3)

We used R and RStudio with the following packages (tidyverse-v1.2.1, ggplot-v3.5.2, gt-v1.0.0, gtsummary-v2.1.0, and ggsignif-v0.6.4) to clean, combine, explore, visualize and summarize our data sets, see supplemental section for software and data references.(7–12) We then filtered the COI data set for the year 2020, following COI use guidelines, joining COI observations with our data set based upon matching zip codes. International patients did not have a COI. Most data presented is descriptive except for assessing the modes of death by race and COI numbers. A Fisher's exact test or a Kruskal-Wallis rank sum test was applied respectively to determine mode of death differences between race & ethnicity and COI. For investigation of the COI by race and ethnicity, we applied an ANOVA and TukeyHSD to determine significant differences between multiple groups.

References

1. Child Opportunity Index (COI) | diversitydatakids.org [Internet]. Available from: https://www.diversitydatakids.org/child-opportunity-index

2. Acevedo-Garcia D, Noelke C, McArdle N, Sofer N, Hardy EF, Weiner M, et al. Racial And Ethnic Inequities In Children's Neighborhoods: Evidence From The New Child Opportunity Index 2.0. Health Affairs (Project Hope). 2020 Oct;39(10):1693–701.

3. COI 3 Technical Documentation | diversitydatakids.org [Internet]. Available from: https://www.diversitydatakids.org/research-library/research-report/coi-30-technical-documentation

4. Census bureau data [Internet]. Available from: https://data.census.gov/

5. HPSA find [Internet]. Available from: https://data.hrsa.gov/tools/shortage-area/hpsa-find

6. MUA find [Internet]. Available from: https://data.hrsa.gov/tools/shortage-area/mua-find

7. R: The r project for statistical computing [Internet]. Available from: https://www.r-project.org/

8. Posit team. RStudio: Integrated development environment for r. [Internet]. Boston, MA: Posit Software, PBC,; 2024. Available from: http://www.posit.co/

9. Wickham H, Averick M, Bryan J, Chang W, McGowan LD, François R, et al. Welcome to the Tidyverse. Journal of Open Source Software [Internet]. 2019 Nov 21;4(43):1686. Available from: https://joss.theoj.org/papers/10.21105/joss.01686

10. Ahlmann-Eltze C, Patil I. Ggsignif: R package for displaying significance brackets for 'ggplot2'.

11. Iannone R, Cheng J, Schloerke B, Hughes E, Lauer A, Seo J, Brevoort K, Roy O. Gt: Easily create presentation-ready display tables [Internet]. 2024. Available from: https://github.com/rstudio/gt

12. Sjoberg DD, Whiting K, Curry M, Lavery JA, Larmarange J. Reproducible Summary Tables with the gtsummary Package. The R Journal [Internet]. 2021;13(1):570–80. Available from: https://journal.r-project.org/archive/2021/RJ-2021-053/index.html
